# A multi-institutional study of the prevalence of *BRCA1* and *BRCA2* large genomic rearrangements in familial breast cancer patients

**DOI:** 10.1186/1471-2407-14-645

**Published:** 2014-09-01

**Authors:** Moon-Woo Seong, Sung Im Cho, Kyu Hyung Kim, Il Yong Chung, Eunyoung Kang, Jong Won Lee, Sue K Park, Min Hyuk Lee, Doo Ho Choi, Cha Kyong Yom, Woo-Chul Noh, Myung Chul Chang, Sung Sup Park, Sung-Won Kim, Korean Hereditary Breast Cancer Study Group

**Affiliations:** Department of Laboratory Medicine, Seoul National University Hospital, Seoul, Korea; Department of Surgery, Seoul National University Bundang Hospital, Sungnam, Korea; Department of Surgery, National Medical Center, Seoul, Korea; Department of Surgery, Asan Medical Center, University of Ulsan College of Medicine, Seoul, Korea; Department of Preventive Medicine, Seoul National University College of medicine, Seoul, Korea; Department of Biomedical Sciences, Seoul National University Graduate School, Seoul, Korea; Cancer Research Institute, Seoul National University College of Medicine, Seoul, Korea; Department of Surgery, Soonchunhyang University Hospital, Seoul, Korea; Department of Surgery, Samsung Medical Center, Sungkyunkwan University, Seoul, Korea; Department of Surgery, Myongji Hospital, Goyang, Korea; Korea Institute of Radiological & Medical Sciences, Korea Cancer Center Hospital, Seoul, Korea; Department of Surgery, Dankook University Hospital, Cheonan, Korea

**Keywords:** Breast cancer, Hereditary cancer, Large genomic rearrangement, *BRCA1*, *BRCA2*

## Abstract

**Background:**

Large genomic rearrangements (LGRs) in the *BRCA1/2* genes are frequently observed in breast cancer patients who are negative for *BRCA1/2* small mutations. Here, we examined 221 familial breast cancer patients from 37 hospitals to estimate the contribution of LGRs, in a nationwide context, to the development of breast cancer.

**Methods:**

Direct sequencing or mutation scanning followed by direct sequencing was performed to screen small mutations. *BRCA1/2* small mutation-negative patients were screened for the presence of LGRs using a multiple ligation-dependent probe amplification (MLPA) assay.

**Results:**

Using a combined strategy to detect the presence of small mutations and LGRs, we identified *BRCA1/2* small mutations in 78 (35.3%) out of 221 familial breast cancer patients and *BRCA1* LGRs in 3 (2.1%) out of 143 *BRCA1/2* small mutation-negative patients: the deletion of exons 11–13, the deletion of exons 13–15, and whole gene deletion of exons 1-24. The novel deletion of exons 11–13 is thought to result from a non-homologous recombination event mediated by a microhomology sequence comprised of 3 or 4 base pairs: c.3416_4357 + 1863delins187 (NG_005905.2: g.33369_44944delins187).

**Conclusions:**

In this study, we showed that LGRs were found in 3.7% (3/81) of the patients who had mutations in *BRCA1* or *BRCA2*, and 7.5% (3/40) of patients with mutations in *BRCA1*. This suggests that the contribution of LGRs to familial breast cancer in this population might be comparable to that in other ethnic populations. Given these findings, an MLPA to screen for mutations in the *BRCA1* gene is recommended as an initial screening test in highly selective settings.

## Background

*BRCA1* and *BRCA2* are the two major genes that contribute to the development of hereditary breast cancer. Mutations in these two genes are observed in 15–20% of hereditary breast cancer cases but less than 5% of overall breast cancer cases [1, 2]. Most *BRCA1* or *BRCA2* gene aberrations are small mutations involving a single or multiple changes in nucleotide sequence, but large genomic rearrangements (LGRs) have also been reported in patients who are negative for *BRCA1* or *BRCA2* sequence variations. The prevalence of LGRs varies according to the ethnicity and selection criteria of the study population [3, 4]. In certain studies conducted in the Netherlands and Italy, it was shown that in breast and/or ovarian cancer patients who tested negative for *BRCA1* or *BRCA2* small mutations, about 20% carried an LGR in 1 of these 2 genes [5, 6], but many other studies reported a prevalence of under 10% [7, 8]. Although the prevalence of LGRs is expected to be low in the population in this study compared to other ethnic populations, the findings reported here are based on a limited study and the nationwide prevalence is not known yet in this population [9, 10].

Here, we analyzed 221 familial breast and/or ovarian cancer patients from 37 different hospitals throughout Korea to estimate the contribution of LGRs to the development of breast cancer in a nationwide context.

## Methods

### Subjects

**Figure 1:**
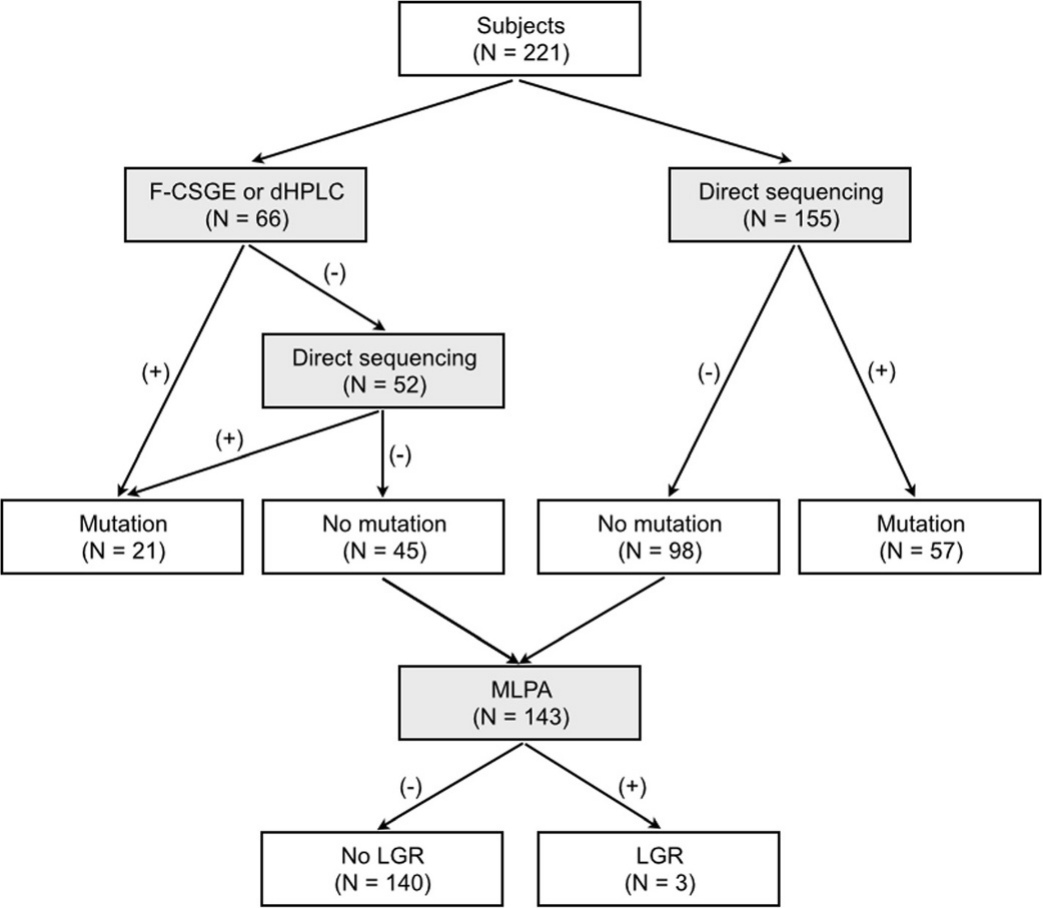
**The study flowchart outlining the number of subjects and the genetic testing approach used in the study.** A total of 221 familial breast cancer patients were included and MLPA analysis was performed for 143 patients who did not have small mutations in the *BRCA1/2* genes.

Patients who fulfilled the following criteria were selected for this study between February 2006 and November 2011: female patients with breast cancer diagnosed at any age, patients with a family history of 2 or more breast cancer cases or 1 or more ovarian cancer cases. In total, 221 patients were enrolled from 37 hospitals throughout the country. The mutational status of the *BRCA1* and *BRCA2* genes was determined for all patients by using the following approaches: direct sequencing (155 patients), or mutation scanning including fluorescence-based conformation-sensitive gel electrophoresis (F-CSGE) and denaturing high-performance liquid chromatography (dHPLC) followed by direct sequencing (66 patients) (Figure 1). In the latter approach, direct sequencing was performed when a patient was found to be positive using the F-CSGE or dHPLC (14 patients) methods; direct sequencing was otherwise carried out for the entire gene (52 patients). Excluding the 78 patients who had mutations identified by any of the above approaches, the remaining 143 patients were screened for LGRs. All patients received genetic counseling and BRCA genetic testing was performed after obtaining informed consent. This study was approved by the institutional review board of Seoul National University Bundang Hospital.

### Mutation screening

Genomic DNA was extracted from peripheral blood samples using Gentra PureGene DNA Isolation Kits (Gentra Systems, Inc. Minneapolis, MN). F-CSGE and dHPLC were performed as previously described [11]. PCR was performed using primers the flanked the splice junctions of the coding exons [12]. Amplified products were sequenced using an ABI 3730 analyzer (Applied Biosystems, Foster City, CA), with Bigdye Terminator v3.1 Cycle Sequencing Kits. The sequences were analyzed using SeqScape software (Applied Biosystems, Foster City, CA) and Mutation Surveyor (Softgenetics, State College, PA).

### Multiplex ligation-dependent probe amplification (MLPA)

*BRCA1* and *BRCA2* LGRs were screened using MLPA. MLPA was performed using the SALSA P002/P002B BRCA1 Kit (MRC Holland, Amsterdam, Holland) for *BRCA1* and P045/P045B BRCA2 Kit (MRC Holland, Amsterdam, Holland) for *BRCA2*. PCR products were analyzed using an ABI 3100 analyzer with Genemarker v1.51 (Softgenetics, State College, PA). Peak heights were normalized, and a deletion or duplication was identified when the normalized peak ratio value was below 0.75 or above 1.30.

### Characterization of *BRCA1*LGR

Long-range PCR was performed with the primers 1 F-5′-GGAACTAACCAAACGGAGCA-3′ and 1R-5′-AGGATTGCTTGAGCCTGAAA-3′ using genomic DNA template isolated from patient samples showing a large deletion in exons 11–13. The PCR cycling conditions were as follows: an initial denaturation at 94°C for 2 min; 10 cycles at 94°C for 15 s, 65°C for 30 s, and 68°C for 7 min; 25 cycles at 94°C for 15 s and 65°C for 30 s; a final extension at 68°C for 7 min (increase of 20 s per cycle). Amplified products were sequenced with additional sequencing primers (4 F-5′-AACCACAGTCGGGAAACAAG-3′, 5 F-5′- TAGGGGTTTTGCAACCTGAG-3′) using an ABI 3730 analyzer (Applied Biosystems, Foster City, CA) and analyzed using SeqScape software (Applied Biosystems, Foster City, CA) and Mutation Surveyor (Softgenetics, State College, PA). The deletion was described using the nomenclature used for sequence variations as recommended by the Human Genome Variation Study ( http://www.hgvs.org/mutnomen); NM_007294.3 was used as the coding DNA reference sequence.

### Statistical analysis

The comparisons between the groups were performed using the results from Mann-Whitney U tests. Statistical data was analyzed using SPSS version 16.0 (SPSS, Inc.).

## Results

**Figure 2:**
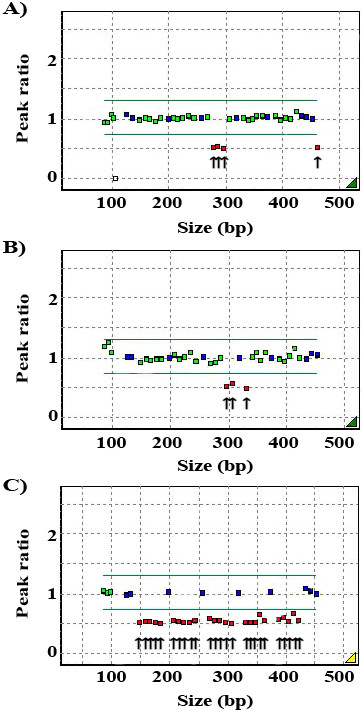
**The 3** ***BRCA1*** **LGRs identified in the study using MLPA screening.** The MLPA analysis demonstrates **A)** exons 11–13 deletion, **B)** exons 13–15 deletion, and **C)** whole gene deletion of exons 1-24. Exons having a reduced peak ratio are denoted with the arrows.

We identified *BRCA1/2* small mutations in 78 (35.3%) out of 221 familial breast cancer patients using direct sequencing, or mutation scanning including F-CSGE and dHPLC followed by direct sequencing, and identified *BRCA1* LGRs in 3 (2.1%) out of 143 *BRCA1/2* small mutation-negative patients using MLPA (Figure 1). In total, the prevalence of *BRCA1/2* mutations was 36.7% (81 of 221) in this study. The prevalence of *BRCA1* mutations (49.4%, 40/81) was slightly lower than that of *BRCA2* mutations (50.6%, 41/81). The three different *BRCA1* LGRs identified in the study were as follows: the deletion of exons 11–13, the deletion of exons 13–15 and a whole gene deletion of exons 1-24 (Table 1; Figure 2).

**Table 1:** **The characteristics of the** ***BRCA1*** **gene in the 3 patients identified with** ***BRCA1*** **LGRs**

Patient	Deleted exons (size)	Nomenclature	Mechanism	Age at diagnosis	Family history	Tumor stage	IHC
P1	Exons 11–13 (11,389 bp)	c.3416_4357 + 1863delins187	*Alu*/non-*Alu* NHE	35	BC+/OC-	0	ER-/PR-/Her2-
P2	Exons 13–15 (11,467 bp)	c.4186-1593_4676-1465del	*Alu*/*Alu* HR	35	BC+/OC+	IIa	ER+/PR+
P3	Whole gene	–	NA	36	BC+/OC-	IIa	ER-/PR+/Her2-

*Abbreviation:* NHE, non-homologous event; HR, homologous recombination; NA, not available; BC, breast cancer; OC, ovarian cancer.

**Figure 3:**
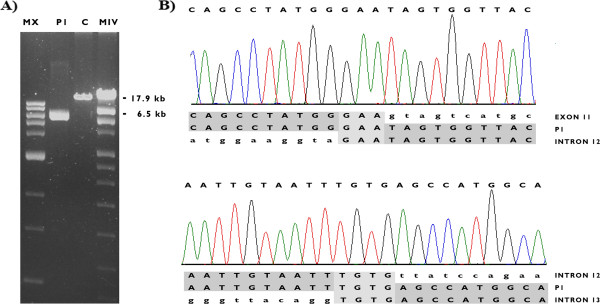
**A detailed characterization of the deletion of exons 11–13 in the** ***BRCA1*** **gene identified in patient P1. A)** Long-range PCR results showing an approximately 11 kb-sized deletion in the *BRCA1* gene; **B)** DNA sequence analysis showing that the deletion in the *BRCA1* gene in this case corresponds to NM_007294.3: c.3416_c.4357 + 1863delins187 [NG_005905.2: g.33369_44944delins187]. The 5′ and 3′ breakpoints in the gene are located within exon 11 and intron 13, respectively, with a partial insertion of intron 12 sequence at the breakpoint (c.4185 + 2603_2789). Short common sequences (microhomology) are shown shaded in light gray between the rearranged sequences at each breakpoint. MX and MIV: molecular weight ladders; P1: patient 1; C: healthy control.

Patient P1, who was diagnosed with carcinoma in situ at the age of 35, carries a *BRCA1* gene with deletions of exons 11–13. She had two second-degree relatives with breast cancer. An immunohistochemistry (IHC) study revealed that her tumor was negative for ER, PR, and HER2. The deletion of exons 11–13 was characterized using long-range PCR and subsequent direct sequencing. The patient was identified as having a novel, 11,389 bp-sized deletion that ranged from the base pair at position 681 of the 3′ end of exon 11 to intron 13: c.3416_4357 + 1863delins187 (NG_005905.2: g.33369_44944delins187) (Figure 3). The 5′ breakpoint was not correlated with any *Alu* element, but the 3′ breakpoint was located within an *AluJo* element in intron 13. Interestingly, a partial sequence (187 bp) from intron 12 was inserted at the deletion site. Both ends of the inserted sequences showed a 3 base pair (GAA) or 4 base pair (TGTG) microhomology with the 5′ and 3′ breakpoints, respectively.

Patient P2, who was diagnosed with stage II invasive ductal carcinoma at the age of 35, carries a *BRCA1* gene lacking exons 13–15 . ER and PR were positive on IHC staining. The patient has 1 first-degree relative with ovarian cancer and 1 second-degree relative with breast cancer. The precise characterization of the deletion breakpoint in this patient has been previously reported; this deletion may be the result of *Alu*-mediated homologous recombination events [9].

Patient P3 was diagnosed with stage II invasive ductal carcinoma at age 36 and does not have a copy of the *BRCA1* gene. The patient has 4 second-degree relatives with breast cancer. An IHC study revealed that PR was positive, and ER and HER2 were negative. The deletion breakpoint is uncharacterized in this patient.

The mean age at diagnosis of the 3 patients with *BRCA1* LGRs was younger than that of patients with non-LGR mutations in *BRCA1/2* (35.3 vs. 40.7) or *BRCA1* (35.3 vs. 36.8), but these differences were not statistically significant.

## Discussion

To the best of our knowledge, this is the first nationwide study reporting a screen for *BRCA1*/*2* LGRs in familial breast and/or ovarian cancer patients in Korea. In this study, 3 LGRs were identified in 3 out of 143 *BRCA1* and *BRCA2* small mutation-negative patients. Each patient had an LGR unique to the family. Furthermore, LGRs were found only in the *BRCA1* gene. The most common mechanism of LGRs found in *BRCA1/2* is known as the *Alu*-mediated unequal homologous recombination, followed by non-homologous events such as *Alu*/non-*Alu* or non-*Alu*/non-*Alu*, and a recombination event between the *BRCA1* gene and a pseudogene [4]. This mechanism occurs because of the high *Alu* density (41.5%) in the *BRCA1* gene, which is 4-fold higher than that in the human genome and 2-fold that observed in the *BRCA2* gene [13, 14]. Between 2 fully characterized LGRs in our study, 1 LGR (a deletion of exons 13–15) might occur because of *Alu*-mediated homologous recombination, and the other (a deletion of exons 11–13) because of non-homologous recombination events that are mediated by microhomology. Non-homologous recombination events frequently result in deletions and short insertions at the site of the deletion [15]. Interestingly, our case showed a relatively long insertion of 187 bp at the deletion site.

The prevalence of *BRCA1* LGRs ranges from approximately 6%–27% of all mutations detected in the *BRCA1* gene; *BRCA2* LGRs play a minimal role in breast cancer [4]. In non-Ashkenazi Jewish families in the United States, *BRCA1* and *BRCA2* LGRs comprise 18% (8 of 44) of all identified mutations; 29% (8 of 28) and 6% (1 of 16) of these mutations occur in the *BRCA1* and *BRCA2* gene, respectively [16]. Among Asians from Singapore, LGRs account for 10% (4 of 40) of all mutations and 10.5% (2 of 19) and 9.5% (2/21) in the *BRCA1* and *BRCA2* gene, respectively [17]. So far, there have only been 2 reports describing *BRCA1* LGRs in this population [9, 10]. In the former study, LGRs constituted 2.5% of all *BRCA1/2* mutations and 6.2% of *BRCA1* mutations and LGRs were rarely detected; LGRs were found only in 1.2% of all *BRCA1/2* gene mutations and 2.3% of *BRCA1* gene mutations. Our results demonstrating that 3.7% (3/81) of patients who have mutations in *BRCA1*/*2* and 7.5% (3/40) of patients who have a mutation in the *BRCA1* gene were higher than those reported previously.

## Conclusions

This nationwide study suggests that the contribution of LGRs in the development of familial breast cancer in the Korean population might be comparable to other ethnic populations and a MLPA screening for mutations in the *BRCA1* gene is recommended as an initial screening test in highly selective settings.

## Abbreviations

DHPLC: Denaturing high-performance liquid chromatography
SSCP: Single strand conformational polymorphism
MLPA: Multiple ligation-dependent probe amplification
LGR: Large genomic rearrangement
F-CSGE: Fluorescence-based conformation-sensitive gel electrophoresis.
